# Intraoperative Neuromonitoring During Resection of Gliomas Involving Eloquent Areas

**DOI:** 10.3389/fneur.2021.658680

**Published:** 2021-06-23

**Authors:** Hao You, Hui Qiao

**Affiliations:** Department of Neurophysiology, Beijing Neurosurgical Institute, Capital Medical University, Beijing, China

**Keywords:** glioma involving eloquent areas, intraoperative neuromonitoring, direct electrical stimulation, awake craniotomy, epilepsy

## Abstract

In the case of resection of gliomas involving eloquent areas, equal consideration should be given to maintain maximal extent of resection (EOR) and neurological protection, for which the intraoperative neuromonitoring (IONM) proves an effective and admirable approach. IONM techniques applied in clinical practice currently consist of somatosensory evoked potential (SSEP), direct electrical stimulation (DES), motor evoked potential (MEP), electromyography (EMG), and electrocorticography (ECoG). The combined use of DES and ECoG has been adopted widely. With the development of technology, more effective IONM tactics and programs would be proposed. The ultimate goal would be strengthening the localization of eloquent areas and epilepsy foci, reducing the incidence of postoperative dysfunction and epilepsy improving the life quality of patients.

## Introduction

Glioma is the most common type of primary intracranial tumors and accounts for ~30% of them. It generally originates from glial or precursor cells and can be characterized by complex genetic background and dismal prognosis. Currently, an optimal management of glioma requires a multidisciplinary approach including surgery, radiotherapy, chemotherapy, and supportive care. Among these treatments, a generally accepted goal of glioma surgery is to achieve maximal safe resection, which reflects the need for both prolonging life and protecting neurological function. For gliomas involving eloquent areas, the latter may be particularly important.

Gliomas involving eloquent areas present a specific subtype of gliomas, invading the cortex or subcortical structures associated with sensory, motor, language, and cognitive functions. Surgical resection of gliomas involving eloquent areas has been a real challenge in glioma treatment. As surgical operations are often accompanied by the risk of instant acute partial injuries to eloquent areas, the balance between maximal extent of resection (EOR) and neurological protection is always unmanageable. For this reason, relevant assistive technologies have been introduced.

Over the past few decades, intraoperative neuronavigation using magnetic resonance imaging (MRI), fluorescence imaging, and ultrasonography have been demonstrated as effective techniques in detecting tumor boundaries. In a previous study, the results showed that the combination of 5-ALA and contrast-enhanced ultrasound significantly improved the EOR compared with conventional strategy (median EOR%, 100 vs. 90) (1). Moreover, a clinical trial by Senft et al. showed that 96% patients receiving iMRI got 100% tumor EOR compared with the 68% in the control group. However, iMRI failed to lower the incidence of postoperative neurological dysfunction (PND) in this study (2). As a result, for surgery of gliomas involving eloquent areas, tumor boundary identification is not enough; accurate brain mapping is also highly recommended.

Regarding the technologies for achieving brain mapping, intraoperative functional neuronavigations are commonly applied in glioma resection, which combine with preoperative functional MRI [blood oxygen level dependent MRI and diffusion tensor imaging (DTI) MRI] (3, 4) to determine the brain functional localization. However, DTI cannot display the entire cortico-subcortical circuits, and the accuracy for exhibiting the anatomic regions depends on the fiber tracking software packages employed (5, 6). Moreover, functional MRI does not distinguish between essential but compensable structures and those having to be retained for functional preservation (7). The brief description of intraoperative imaging techniques is shown in Table 1.

**Table 1:** Brief description of intraoperative imaging and neuromonitoring techniques.

**Modality**	**Advantages**	**Disadvantages**
**Intraoperative imaging techniques**		
Intraoperative navigation	Combines with preoperative fMRI and presents real-time intuitive tumor detection	Time-consuming and inevitably affected by brain drift
Intraoperative MRI	Good at detecting tumor boundaries and avoiding the effects of brain shift	Substantially lengthens operation time and cannot contribute to avoid PNDs
Ultrasonography	Real-time tumor localization detection	Insufficient sensitivity for small size tumor
5-ALA fluorescence	Visualization of tumor cells and more sufficient for high-grade gliomas	GTR was relatively low than iMRI and the incidence of PNDs was higher.
**Modality**	**Description**	
**Intraoperative neuromonitoring**		
SSEPs	Localizing the central sulcus	
Direct electrical stimulation	For localizing eloquent areas during general anesthesia and awake craniotomy and allows minimizing the incidence of PNDs while maximizing the EOR of tumors	
MEPs	For preserving motor function	
ECoG	For monitoring spontaneous and stimulus-elicited epileptiform waves at the same time and show a potential for detecting glioma related epilepsy location	

*PNDs, postoperative neurological deficits; 5-ALA, 5-aminolevulinic acid; GTR, gross tumor rate; SSEPs, somatosensory evoked potentials; PNDs, postoperative neurological deficits; EOR, extent of resection; MEPs, motor evoked potentials; ECoG, electrocorticography*.

Therefore, as a unique intuitive technique, intraoperative neuromonitoring (IONM) has become a valid tool for maintaining indispensable neurological function in glioma resection. The IONM technologies currently adopted for gliomas involving eloquent areas include somatosensory evoked potential (SSEP), direct electrical stimulation (DES), motor evoked potentials (MEP), electromyography (EMG), and electrocorticography (ECoG). Among them, DES has been regarded as the gold standard for real-time detection of the brain function in glioma resection. The brief description of intraoperative neuromonitoring techniques are shown in Table 1.

## Intraoperative Neuromonitoring Techniques in the Resection of Gliomas Involving Eloquent Areas

### Somatosensory Evoked Potential

Since last century, SSEP has been widely used to predict brain injury in the process of spinal cord surgery, such as regional ischemia (8). With the development of technology and equipment, SSEP is now applicable for majority of neurological and vascular surgeries that may cause neurological injury. In the 1970s, SSEP was also a new tool to determine the location of the central sulcus in epilepsy treatment (9). Nowadays, phase reversal of SSEP acts as one of the valid parameters for central sulcus positioning throughout the tumor resections (10). Here is the operational procedure of SSEP.

After opening the dura mater, a strip electrode is placed vertically in the central sulcus as the recording electrode. Stimulating electrodes are deployed near the median nerve or posterior tibial nerve on the opposite side of the tumor to obtain a stable SSEP. Afterward, in order to determine the structural position according to the direction of waveform, the electrode of N20 is set representing the position of postcentral gyrus and that of P22 representing the precentral gyrus. If the waveform was inverted, the central groove sits between those two locations.

Although SSEP has now been a routinely used technique with high accuracy to locate the central trench, SSEP phase reversal technology has some unresolved issues in the resection of glioma with particular structures and features. Accordingly, in the study of Romstöck et al. on the electrophysiological data of 230 patients with tumors in the cerebral sensorimotor region, the accuracy of SSEP phase reversal was low in the resection of large central and postcentral tumors (11).

### Direct Electrical Stimulation

In 1886–1887, Horsley reported the first 13 cases of cortical resection by using cortical stimulation (12). However, at that time, there were considerable insufficiency and serious side effects with cortical DES, such as the epilepsy during the surgery.

Nowadays, DES specifically referred to the application of electric current directly onto the cerebral cortex and subcortex for localizing functional regions during craniotomy under general anesthesia or awake. The stimulation modes of DES comprise single-pulse electrical stimulation and high-frequency multi-pulse electrical stimulation. For the purpose of brain mapping, intraoperative monitoring physicians prefer observing the patients' reaction to the electrical stimulation. Electromyography (EMG), direct cortical-stimulated motor-evoked potentials (dcMEPs) and subcortical-stimulated MEPs (scrtMEPs) were mainly applied as recording measures. Among them, dcMEPs and scrtMEPs prove effective for preserving motor function by predicting the edges of the corticospinal tracts (CSTs) (13).

During the resection of glioma in function areas, the intraoperative localization of the functional structural boundary is critical for the protection of neurological function. Due to the individual discrepancy in anatomy and neurological function, relying on anatomical landmarks to locate the function areas may be unreliable. In contrast, intraoperative direct electrical stimulation technology can ensure the realization of real-time monitoring of eloquent areas in cortex and subcortical pathways, which is accurate, reliable, and safe. So far, the effectiveness of DES in preserving neurological function in the excision of low-grade glioma (LGG) via guaranteeing the maximal EOR has been confirmed (14–16). In 2018, a retrospective cohort study of Zhang et al. revealed that glioma patients undergoing IONM (especially the patients with high-grade astrocytic tumor) had longer OS and lower rate of neurologic deficits (17). Similarly, Pan et al. reported that patients receiving tailored IONM had better postoperative KPS grade (mean KPS 81.1 vs. 70.4) and tended to get lower PND rate, although the EOR and OS were not improved (18). DES has become a major and vital intraoperative monitoring method in tumor excision of glioma involving eloquent areas. Moreover, it was also the only way to monitor the function of the language brain region and detect the scope of the language brain area in real time. Intraoperative DES technology uses different protocols under different anesthesia strategies.

#### Anesthesia

In recent years, with the development of anesthesia technology, awake brain tumor surgery has grown as an emerging effective strategy to protect brain function. At the end of the 19th century, Cushing first introduced the term “regional anesthesia” to neurosurgery. Different from local anesthesia, regional anesthesia refers to the application of local anesthesia to the subcutaneous area for nerve blocks, in which advent has helped promote the application of awake neurosurgery. Currently, the anesthesia techniques used for awake craniotomy were asleep–awake–asleep (SAS), monitored anesthesia care (MAC) and awake–awake–awake technique (AAA). Among them, SAS and MAC techniques were commonly used in awake craniotomy and reported to be feasible and safe (19). Lately, AAA technology has emerged as it allows patients to maintain maximum alertness during awake craniotomy and benefits IONM (20).

Now, the combination of remifentanil and/or propofol and other short-acting opioids are commonly employed for craniotomy. Recently, dexmedetomidine (DEX), a selective agonist of α2-adrenergic receptors, proved more desirable for awake craniotomy due to its short arousal time and low probabilities of respiratory depression (21, 22).

#### Direct Electrical Stimulation in Awake Patients

In the early 20th century, Sherrington et al. pioneered the study of brain mapping for motor cortex localization. At the same time, Foerster reported the application of ECoG in epilepsy surgeries. Then, Penfield developed this strategy to the research of language brain area (23).

Nowadays, the intraoperative cortical and subcortical DES acts as a gold standard for localization of eloquent areas of the brain (24, 25). By stimulating the cortex and subcortical structures with appropriate intensity of current, the neurons and conduction bundles are depolarized. Then, the local neural tissues are activated or inhibited, causing the excitement or inhibition of patient's corresponding neurological function.

At present, according to the guidelines for surgical resection of gliomas in functional regions during awake craniotomy published in 2018 by Gliomas Professional Committee of Chinese Medical Doctors Association, direct cortical and subcortical electrical stimulation is recommended to be performed under the real-time monitoring of ECoG to ensure the operational safety and effectiveness (26).

**Figure 1:**
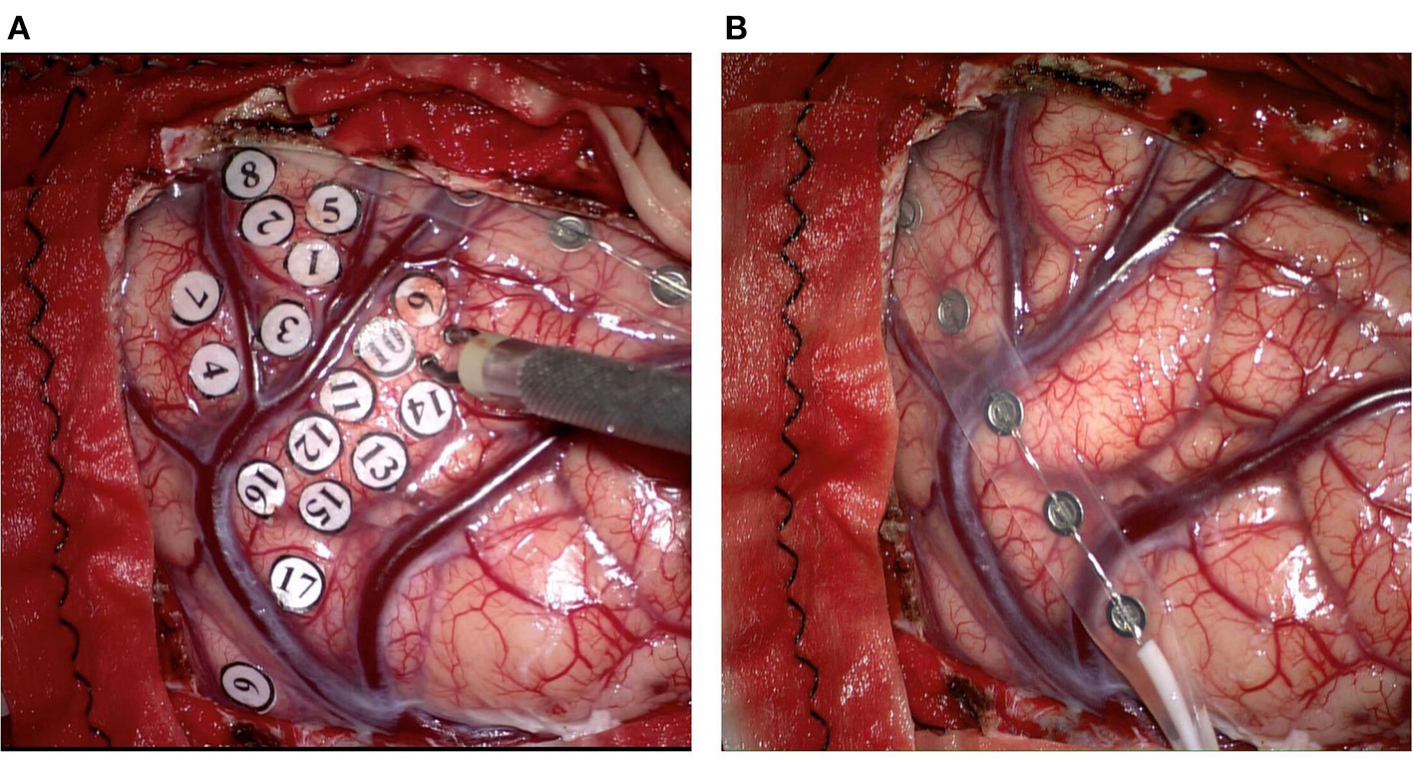
The techniques applied for intraoperative direct electrical stimulation in resection of glioma. **(A)** The Ojemann cortical stimulator which is bipolar probe is applied for localizing eloquent areas to protect essential neurological function. **(B)** A grid electrode containing six electrodes is applied for localizing the central sulcus, and monitoring spontaneous and stimulus-like epileptiform waves in order to find and deal with the epilepsy foci related to tumorigenesis.

Given the variability of optimal stimulus parameters and after-discharge threshold (27), the stimulus intensity varies from person to person with the initial stimulus intensity usually set to 1 mA, and gradually increased by 0.5–1 mA, until a functionally positive stimulation reaction or an after-discharge appears. The after-discharge is generated by direct stimulation of the cortex, which is defined as the maximal stimulus intensity, showing a diffuse epileptic wave on ECoG. For cortical stimulation, bipolar electrical nerve stimulation holds parameters as a 5 mm of bipolar spacing, 50–60 Hz of frequency and 0.8–1 ms of wave width (Figure 1A). DES generally applies continuous stimulation pattern, and at least three times of stimulation are performed sequentially in each target area to confirm the localization of functional regions. In practice, the duration of each stimulus might change with the requirement of specific missions; however, the longest duration should be set at 6 s (26). The internationally recognized parameters for the electrical stimulation are pulse width, 0.1–0.3 ms; stimulation duration, 2–5 s; frequency, 50 or 60 Hz; maximum stimulation current, lower than 20 mA (28).

##### Brain Mapping of Motor and Sensory Functional Area

The upper limbs and face on the opposite side of the stimulus point are exposed completely for the brain mapping of the motor functional area. The positive reactions of the motor functional area were appearing as the involuntary movement of the contralateral limbs or corresponding part of the face, along with the electromyography (EMG) response. However, due to the awake condition, the voluntary movement could interfere with EMG and MEP. Therefore, the activity of the muscle opposite to the surgical site is also recorded as criteria. Moreover, the constant monitoring of MEP could predict the injury of some critical vessels in insular and deep temporal regions, which has now been a useful assistive strategy for motor functional mapping (28, 29). The positive response of sensory functional area manifests as the pulsed abnormal sensations, such as burning and numbness on the opposite limb or head. Also, stimulations on sensory areas sometimes cause body movement as well.

The monopolar stimulation is preferable for identifying corticospinal tract due to the more radiant electric field properties of monopolar probe compared with the bipolar probe and reported to be effective in insular glioma resections (29, 30). Recently, a triple brain mapping method including transcranial, monopolar, and bipolar stimulation has been proposed for locating the motor functional area more accurately (31). As a result, the combined application is considered as superior in preserving motor function and enhancing EOR.

##### Brain Mapping of Speech and Cognition Functional Area

Counting and picture naming represent the language and cognitive functions, respectively, which are performed for the speech and cognition functional area mapping. As for the picture-naming task, the patients first familiarize a set of pictures 1 day before the operation. During the DES on the language functional area, a picture would be displayed for 4 s after every single electrical stimulus, and then the patient is asked to name the picture immediately. The electrical stimulation should be given after the presentation of a new picture and the naming of the picture by patients to avoid occasional conditions such as non-convulsive seizure (32). In this session, patient's compliance and the cooperation between patients and surgical team are important. Too long stimulation or too short interval between stimulations may cause intraoperative epilepsy. Therefore, continuous stimulations in a short period of time should be avoided. After every single effective stimulus, the corresponding location of the stimulus area is marked by using a sterile label of number.

The advantages of awake DES can be summarized as follows: First of all, the patient stays awake, able to describe the feelings during the whole operation. Thus, the reliability of the localization could be confirmed and recognized by both surgeons and patients. Second, the monitoring of the brain regions with suppression functions could be completed in awake craniotomy. Finally, the method could be applied for language brain area. The range of important language functional areas in the cerebral cortex was generally small (<2 cm^2^), and the specific anatomical localization often shows great individual differences. Besides, since the response of language functional area to stimulation has always been inhibited, direct cortical and subcortical electrical stimulation techniques under awake anesthesia stand absolutely essential.

#### Direct Electrical Stimulation in Patients Under General Anesthesia

Currently, a combination of a single stimulus or a short train by a strip electrode and intraoperative MEP or SSEP monitoring has been recommended for applying DES under general anesthesia. In the last century, the Penfield technique as a standard for direct cortical stimulation adopted the single-pulse stimulus with the current frequency of 50–60 Hz. Since 1993, the multi-pulse short-train stimulation technology with one to five pulses was introduced as a more efficient and safe method, which caused a lower incidence of seizure (33–35). As for the recording manners, MEP and EMG monitoring were used in DES under general anesthesia. MEP responses were recorded by needle electrodes placed subcutaneously in the abductor pollicis brevis and abductor hallucis muscles of the corresponding sides opposite to cerebral lesion locations. MEP recording interval depended on both the stimulus interval and the relative locations of the lesions to the brain eloquent areas. In a previous research, 53 patients with gliomas involved in the eloquent area who got craniotomy surgery under general anesthesia had less blood loss, mean operative time, and shorter postoperative hospital stays, compared with awake craniotomy (36). However, with the development of anesthesia and electrophysiological technology, DES in awake craniotomy has shown great benefits in real-time monitoring and protecting the neurological function of the language area.

#### Direct Electrical Stimulation in Brain Plasticity Research

Functional remodeling has been recognized in recent years. For patients with diffuse low-grade glioma, due to the relatively slow growth of tumor, the brain functions tend to be modified (37). DES reflects not only the real-time neurological function but also the brain functional plasticity in cases of multiple glioma resections. A study by Picart et al. performed intraoperative cortical and subcortical DES on glioma patients undergoing repeat awake surgery and revealed an optimized EOR and nerve function due to brain plasticity (38).

### Electrocorticography

The first invasive EEG monitoring was performed by Penfield (39) for localizing the epilepsy origin (39), which initiated the EEG monitoring and improved the resection of epilepsy focus and tumors significantly.

With the development of IONM equipment and technology, ECoG could monitor spontaneous and stimulus-elicited epileptiform waves at the same time (Figure 1B). In this process, preoperative routing-electroencephalogram (REEG) or video-EEG (VEEG) examinations were used to identify whether the epileptiform waves originated from an epileptic focus in tumors or the peritumoral areas. In order to find and treat the epilepsy foci related to tumorigenesis, ECoG should be monitored before and after the tumor resection. If the epileptiform waves are still present in the corresponding part after the resection, treatment such as fulguration could be performed in focal areas. Once the intraoperative seizures were detected by ECoG, the electrical stimulation should be stopped immediately, and the cold Ringer irrigation or intravenous injection of antiepileptic drugs was carried out (40–43). The current intensity of DES is controlled below a certain threshold to avoid the intraoperative epilepsy and false-positive consequences of electrical stimulation of the cortex. After-discharges could also be generated by electrical stimulation of the cortical areas, which once detected, indicates that the current intensity should not be elevated any more. However, as the threshold of after-discharges varies with the individual or the cortical region (44), the current intensity, duration, and repetition rate of stimulation should be adjusted according to actual situations.

Nowadays, the most commonly used equipment for ECoG monitoring involves intracranial strip and grid electrodes (45, 46). Yet, considering the number and arrangement of electrodes, there are several restrictions on the application of the equipment. Recently, with the development of biomedical engineering and materials science, Karim et al. had developed a so-called “circular grid” electrode, which could enable tumor resection and 360° monitoring of cortical discharge activity at the same time. Compared with traditional strip electrodes, this state-of-the-art technique retains several advantages such as more accurate intraoperative epilepsy detection, larger resection rate, and better functional outcomes (47).

## Conclusion

In conclusion, the resection of glioma involving eloquent areas aims to both protect the neurological function and ensure the maximal EOR. In the last few decades, the effectiveness of intraoperative monitoring to protect nerve function has been demonstrated. In recent years, awake craniotomy with simultaneous monitoring of multiple electrophysiological indicators has become a tendency. DES and ECoG monitoring are carried out at the same time by most neurosurgeons and electrophysiologists. With the development of further research, more complete and effective intraoperative monitoring approaches would be proposed to propel localizing the eloquent areas and epilepsy foci, reduce postoperative dysfunction, or postoperative epilepsy, through which the life quality of patients would be improved substantially.

## Author Contributions

HY carried out the collection of publications and drafted the manuscript. HQ carried out the review and proofreading of the manuscript. All authors read and approved the final manuscript.

## Conflict of Interest

The authors declare that the research was conducted in the absence of any commercial or financial relationships that could be construed as a potential conflict of interest.
